# Clinical implications of intratumor heterogeneity: challenges and opportunities

**DOI:** 10.1007/s00109-020-01874-2

**Published:** 2020-01-22

**Authors:** Santiago Ramón y Cajal, Marta Sesé, Claudia Capdevila, Trond Aasen, Leticia De Mattos-Arruda, Salvador J. Diaz-Cano, Javier Hernández-Losa, Josep Castellví

**Affiliations:** 1 Translational Molecular Pathology, Vall d’Hebron Institute of Research (VHIR), Universitat Autònoma de Barcelona, Passeig Vall d’Hebron 119-129, 08035 Barcelona, Spain; 2 Pathology Department, Vall d’Hebron Hospital, Passeig Vall d’Hebron 119-129, 08035 Barcelona, Spain; 3 Spanish Biomedical Research Network Centre in Oncology (CIBERONC), Barcelona, Spain; 4 Department of Pathology, Vall d’Hebron University Hospital, Autonomous University of Barcelona, Pg. Vall d’Hebron, 119-129, 08035 Barcelona, Spain; 5 Department of Genetics and Development, Columbia University Medical Center, New York, NY 10032 USA; 6 Vall d’Hebron Institute of Oncology, Vall d’Hebron University Hospital, c/Natzaret, 115-117, 08035 Barcelona, Spain; 7 Department of Histopathology, King’s College Hospital and King’s Health Partners, London, UK

**Keywords:** Intratumor heterogeneity, Liquid biopsy, Artificial intelligence, Antitumor therapeutics

## Abstract

In this review, we highlight the role of intratumoral heterogeneity, focusing on the clinical and biological ramifications this phenomenon poses. Intratumoral heterogeneity arises through complex genetic, epigenetic, and protein modifications that drive phenotypic selection in response to environmental pressures. Functionally, heterogeneity provides tumors with significant adaptability. This ranges from mutual beneficial cooperation between cells, which nurture features such as growth and metastasis, to the narrow escape and survival of clonal cell populations that have adapted to thrive under specific conditions such as hypoxia or chemotherapy. These dynamic intercellular interplays are guided by a Darwinian selection landscape between clonal tumor cell populations and the tumor microenvironment. Understanding the involved drivers and functional consequences of such tumor heterogeneity is challenging but also promises to provide novel insight needed to confront the problem of therapeutic resistance in tumors.

## Background

Malignant tumors have highly diverse phenotypic and molecular characteristics both at the intertumor and intratumor levels [1]. Intertumor heterogeneity (also known as interlesion heterogeneity) refers to the differences found between tumors in different patients. Intratumor heterogeneity (also known as intralesion heterogeneity) refers to distinct tumor cell populations (with different molecular and phenotypical profiles) within the same tumor specimen [1].

Cancer is typically defined as a genetic disease driven by oncogenic mutations. In a similar gene-centric view, intratumoral heterogeneity has traditionally been attributed to genetic diversity within cancer cell populations. However, recent evidence suggests that a tumor is heterogeneous in almost every discernible phenotypic trait as the result of not only genetic influences but also non-genetic sources of variability [2]. These non-genetic influences shape the phenotypic states of cancer cells at the proteome level. These factors are the primary determinants of the identity of most cell types in healthy tissue, given that most cells, while phenotypically different, share the same genetic load.

Tumor heterogeneity is associated with poor prognosis and outcome [3–6]. It is thought that intratumor heterogeneity is one of the leading determinants of therapeutic resistance and treatment failure and one of the main reasons for poor overall survival in cancer patients with metastatic disease [1, 7]. Tumors are composed of mosaics of cancer cells with different characteristics and varying sensitivities to anticancer therapies. Tumor heterogeneity has differential layers of complexity. Because cancer is a heterogeneous dynamic target, individual patients, lesions, and cell populations should be thoroughly characterized at varying times. Tumor heterogeneity has presented a considerable challenge to matching patients with the right treatment at the right time; therefore, it poses a challenge to accomplish the goals of precision medicine [8, 9].

It has been shown that most of the targets considered as druggable and for which Food and Drug Administration (FDA)-approved therapeutics are available are not expressed in a uniform or homogeneous manner in tumor tissue. Examples include the variable threshold of positivity for the expression of the HER2 receptor in gastric adenocarcinoma [10], progesterone and estrogen receptors in breast tumors [11], the EML4-ALK translocation in lung adenocarcinoma [12], B-RAF mutations in melanoma, and the 1p/19q allelic loss in oligodendrogliomas [13, 14]. This variability means that many patients undergo specific therapeutic regimens in situations where perhaps only 10% of the studied tumor cells are positive for the corresponding target. Thus, targeted therapies in heterogeneous neoplasms lead to transient tumor regression and subsequent selective outgrowth of existing resistant populations, leading to recurrence in the long run.

In the following sections, we discuss intratumor heterogeneity and in particular how this is driven at the genetic level and in relation to the microenvironment, and how this translates into specific phenotypes at the functional level. Finally, we discuss research approaches needed to advance our understanding of this complex biological phenomenon and how this can lead to novel therapeutic approaches.

## Phenotypic heterogeneity: the particular case of morphologic heterogeneity

Every tumor is unique as a result of its interactions with the host and the genetic and epigenetic variability. This results in important differences between tumors from different individuals, named as intertumoral heterogeneity. At this level, we recognize more than 250 types of tumors with distinctive clinical-pathological characteristics and that show most of them also peculiar pathological characteristics. In most of these tumor types, dozens or hundreds of pathological variants are observed. In such variability of tumors and, therefore, of types of cancer, factors such as the location and the cell type are determinant.

**Fig. 1:**
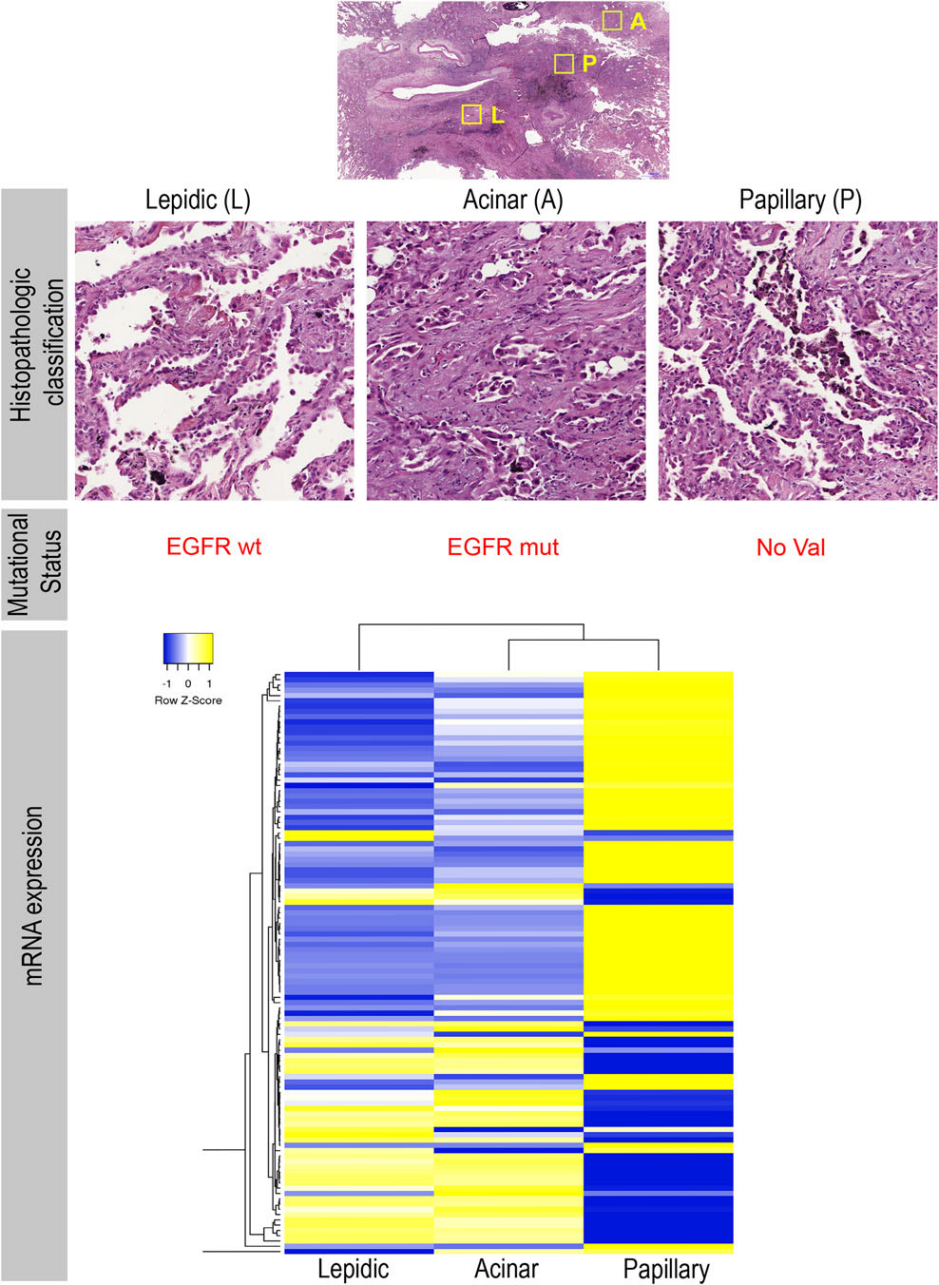
Lung cancer intratumoral heterogeneity at morphological and molecular levels. **a** Paraffin section of a lung tumor biopsy showing three main morphological subtypes within the same tumor. **b** Molecular and biomarker analysis confirming heterogeneity in EGFR mutation and in the **c** transcriptional signature of these three subtypes

Neoplastic lesions are diagnosed primarily based on pathological examination, both gross and microscopic, but the information obtained may not always be conclusive for a diagnosis of malignancy. In this context, intratumoral heterogeneity poses an unresolved problem. Most carcinomas, sarcomas, and astrocytomas display extensive intratumoral morphological variability that, if not taken into account, can lead to an inaccurate or even incorrect diagnosis. For example, analysis of complete specimens of lung adenocarcinoma often reveals more than one morphological pattern (acinar, solid, lipid, papillary, micropapillary, mucinous, or pleomorphic) (Fig. 1) [15], and accurate assessment is critical for the right diagnosis and prognosis. Therefore, diagnosis based on morphology requires extensive areas of the tumor to be examined to ensure an objective, genuinely representative snapshot of the heterogeneity within the tumor as a whole. State-of-the-art digital image acquisition and quantification algorithms, which integrate biophysical parameters to capture the spatial variation in tumor architectures, are likely to play an essential role in this [16].

In many malignant tumors, it is common to find well-differentiated areas adjacent to poorly or moderately differentiated areas. Attempts are currently being made to quantify these areas and grade tumors, generally according to the least-differentiated area or the area with the highest degree of cytologic malignancy [17, 18]. This intratumoral differentiation is often patchy, and not well defined in molecular terms. It reproduces the development patterns and morphofunctional specialization present in the tissue where cancer originated and can lead to differences in the expression of some of the therapeutic targets and, therefore, the response to a specific treatment [10, 19–21]. One of the most characteristic examples is EGFR-driven lung adenocarcinoma in non small-cell lung cancer (NSCLC), as there have been cases of resistance associated with conversion to small-cell lung cancer (SCLC) phenotype after long-term treatment with EGFR tyrosine kinase inhibitors [22].

Similarly, the observation of specific morphologic patterns in human tumors has made it possible to identify distinct genetic changes. For example, characteristic chromosomal translocations have been identified in round cell desmoplastic tumors, clear cell sarcoma, synovial sarcoma, and rhabdoid tumors [23, 24]. Nevertheless, not all oncogenic changes are diagnostic determinants of a specific tumor type or give rise to the emergence of a specific morphologic pattern. Researchers from the National Research Tomsk State University [25] have shown that the morphological heterogeneity in invasive micropapillary carcinoma (IMPC) of the breast is not related to the presence of specific chromosomal aberrations. This heterogeneity responds to specific gene expression profiles, thus pointing to the existence of other determinants of intratumor morphological heterogeneity and highlighting the importance of context. Furthermore, some of the most common molecular alterations have been associated with tumors whose morphological characteristics are strikingly distinct.

## Molecular heterogeneity: a genomic substrate for both tumor biology and evolution

During the 1990s, and after the discovery of oncogenes, it was thought that specific genetic changes would account for tumor heterogeneity and the emergence of an eventual phenotype of resistance to many conventional treatments.

Nevertheless, the puzzle and variability of cancer pathology are made tremendously complicated at the genomic level by the vast number of DNA changes, with thousands of known translocations and more than 1500 mutations, deletions, and amplifications reported to date [8, 26–29]. Also noteworthy is the complex world of microRNAs (miRNAs), of which there are thousands described (www.mirbase.org) and which can act like oncogenes or tumor suppressors (oncomiRs and tumor suppressor miRs) [30, 31], and the unknown role of long non-coding RNA (lncRNA), numbering as many as 60,000 loci in the human genome [32–34]. Importantly, there are many nonspecific genetic alterations in human tumors. For example, the ETV6-NTRK3 translocation can be detected in very different types of tumors such as infantile fibrosarcoma, cellular mesoblastic nephroma, and secretory carcinoma of the breast [35]. Chromosomal translocations such as those including the ALK gene are demonstrated in anaplastic lymphoma, lung adenocarcinoma, and myofibroblastic tumors [36], and the translocation EWSR1-CREB1 in tumors as different as clear cell sarcoma and angiomatoid fibrous histiocytoma [37]. BRAF mutations and translocations have been described in melanocytic nevi, malignant melanoma, colon adenocarcinoma, glioblastoma and pilocytic astrocytoma [38, 39] and EGFR mutations, amplifications in lung adenocarcinoma and brain tumors [40–42].

Although some molecular alterations are recurrent in some tumors, not all the tumors of the same type, and similar morphology, show the same genetic profile. In fact, there is a huge intertumoral heterogeneity between tumors with the same histology in different individuals. This heterogeneity poses a problem when trying to standardize a therapy in a specific tumor type, requiring a personalized approach based on the particular genetic alterations of the tumor, to improve the response rates to the treatment.

Moreover, initial studies have shown that a characteristic morphological pattern could be due to specific oncogenic changes and that malignancy is dependent on the immune response [43]. For example, primary cells with RAS, NEU, mutated p53, MYC, and the viral gene E1A oncogenes injected into athymic mice [43] form different morphological patterns in melanoma [17]. In this sense, the first point to underline would be that the presence of different morphological patterns within the same tumor suggests the coexistence of various clones, each subject to specific genetic changes or different environmental pressures that are not necessarily shared by clones present in other areas.

Critically, this genetic variability is also thought to occur extensively within a tumor (Fig. 1). Therefore, the goal of preparing selective oncograms with chemotherapy drugs and other inhibitors has become complicated—almost impossible—following conventional strategies. Intratumor molecular heterogeneity, and more specifically that occurring intratumorally, is thought to account for the unsuccessful attempts of pharmacologic and radiologic cancer treatments. In fact, in cell lines, a correlation has previously been shown between resistance to radiotherapy and cytotoxic drugs (e.g., cisplatin, doxorubicin, and taxanes) and the expression of specific oncogenes, principally, RAS, p53, c-MYC, and the adenoviral gene E1A [44–47].

### Causes of molecular heterogeneity

**Fig. 2:**
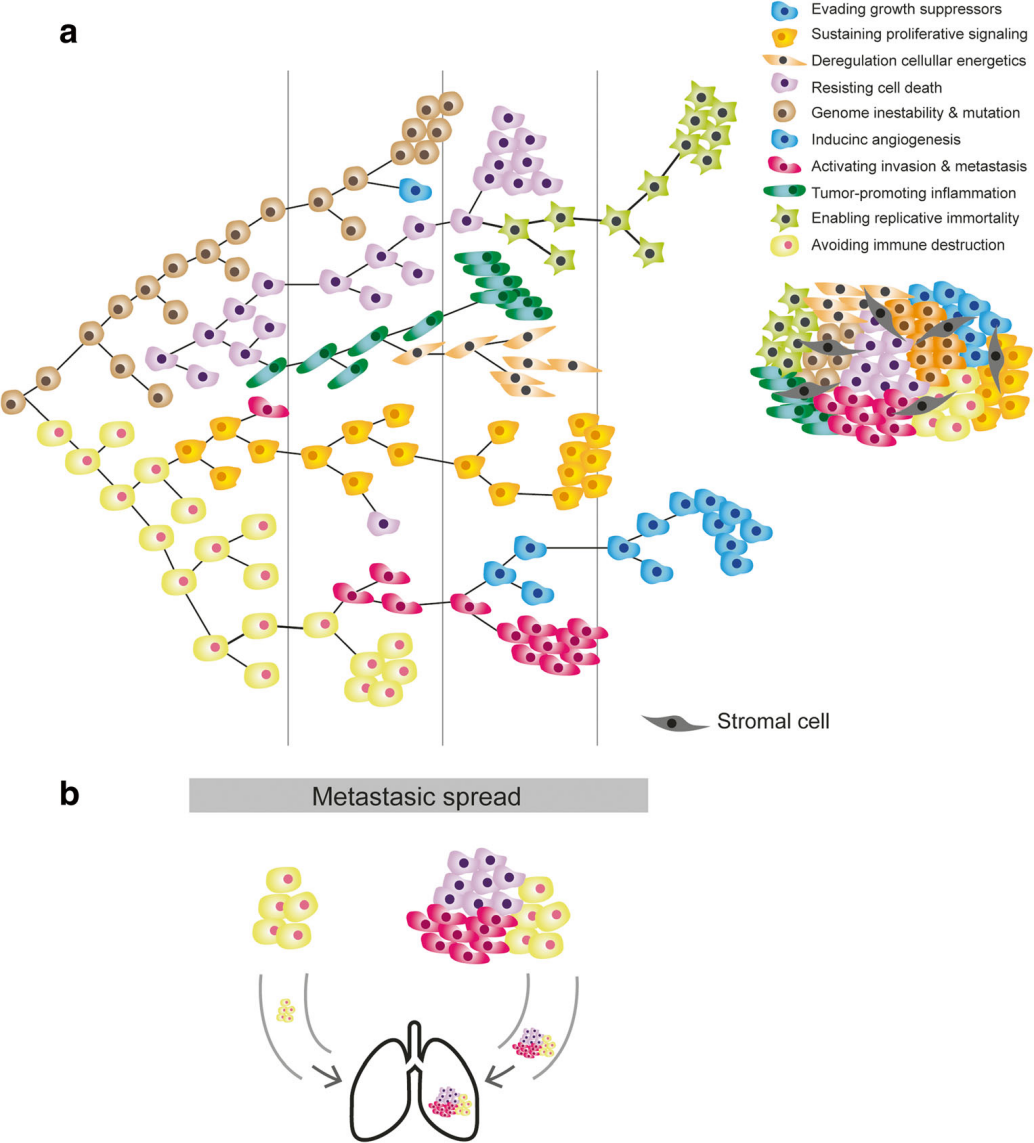
Clonal cooperation and cellular consortium. **a** Darwinian model of clonal heterogeneity resulting in a consortium of clones, each with their characteristics and malignant features. **b** Cooperation between several clones to invade and metastasize

There are a lot of redundant genetic alterations in cellular and biological pathways. These pathways can be grouped into specific “hallmarks” or basic principles that rationalize tumor biology and that are altered in the vast majority of malignant neoplasms [48, 49]. Authors have postulated that the most important aspect of tumor transformation and subsequent progression is the functional alteration of at least ten major biochemical pathways. Given that phenotypes generated by changes in genetic material are the substrate of clonal development and selection and adaptation to the microenvironment for each of these pathways (e.g., insensitivity to apoptosis, self-sufficiency in cell proliferation, acquisition of so-called replicative immortality), several significant genetic changes must occur (Fig. 2) [62]. A representative example can be seen in signaling in the Ras/MAPK and PTEN/PI3K/AKT axes in lung adenocarcinomas, where specific mutations, amplifications, gene expression, and translocations in membrane receptors, as in other genes downstream, can enable the tumor cell hallmark of uncontrolled proliferation [60, 63].

This concept can be extrapolated to other significant pathways, whose number is expected to grow to 15–20 in the coming years [49]. Although “single hit pathways” are reported, disruptions of several pathways are necessary for a cell to become malignant. Moreover, a similar biological effect can be achieved by hitting a particular biochemical pathway at different points, often driven by the inherent genetic instability of a tumor, thus making cancer an extremely heterogeneous (and redundant) disease at the molecular level [54, 64, 65] (Fig. 1).

### Understanding the role of genomic instability as an enabling characteristic of cancer

Despite claims in recent models that only three driver mutations are required for the development of various forms of advanced cancer [66, 67], the number of molecular changes necessary to enable the emergence of a clinically relevant tumor has, for some time, been assumed to be higher than previously thought, given standard mutation rates in any cell type. This observation is supported by the large number of somatic mutations and epigenetic alterations found in most tumor specimens (in the order of thousands), which points to the existence of molecular mechanisms that enable the mutational landscape of cancer cells to expand. Therefore, to explain the substantial molecular variability inherent to malignant tumors, there must be a background of replicative immortality and genomic instability, which is associated with abnormalities in DNA repair mechanisms and maintenance of genetic and chromosomal integrity (see review [68]). Chromosome instability (CIN) and microsatellite instability (MSI) have been described as two alternative pathways to cancer [53, 69, 70].

## Epigenetic heterogeneity

Genetically identical cell populations can display remarkable morphological diversity. One mechanism by which different environmental stimuli drive such heterogeneity is by epigenetic modifications of the genome, which can persist over many cell divisions [61]. Therefore, within what we understand as molecular heterogeneity, regional differences in epigenetic status have been observed in different types of cancer that can act, in much the same way as genetic alterations, as drivers of the tumor process. For example, in colon adenocarcinomas, a subtype harbors a relevant profile of epigenetic alterations [64]; this is also seen in urological [65] and other tumors [57, 58, 71]. Moreover, local hypoxia may induce the expression of histone demethylases and other epigenetic modifiers that subsequently modulate the expression of genes linked to a specific phenotype (e.g., leading to epithelial to mesenchymal transition). Inflammatory cytokines are another example, released by stromal or immune cells, which can alter DNA methylation and other epigenetic markers.

Many of these regional epigenetic differences are associated with an aberrant methylation pattern in specific promoters or other regulatory elements causing either gene activation or silencing [59, 72–77], which may also be predictive of the phylogenetic relationships between the different clones in tumors such as prostate cancer [78]. Therefore, we can deduce that genomic and epigenomic diversity are not mutually exclusive but can be explained by a unified evolutionary process, giving rise to more robust evolutionary models than clonal relationships inferred from genetic or epigenetic datasets alone. A summary of main molecular events related with intratumoral heterogeneity is shown in Table 1.

**Table 1:** Table summarizing the aspects highlighted in this review correlating with the key molecular events related with intratumoral heterogeneity

	Key points	Bibliography
1. Phenotypic heterogeneity	1.1. Hundreds of tumor types and thousands of subtypes	Jamal-Hanjani et al. (2015) [1]
	1.2. Different degree of cell differentiation (low-grade and high-grade tumor types)	Park et al. (2010) [18] Zhou et al. (2015) [17]
	1.3. Morphologic pattern association with genetic changes	Sequist et al. (2011) [22] Nielsen et al. (2015) [24] Zack et al. (2013) [26]
	1.4. Morphological heterogeneity in metastasis vs. primary tumor	Maddipati et al. (2015) [50] Hong et al. (2015) [51]
2. Molecular heterogeneity	2.1. Intratumor heterogeneity and resistance to treatments	Dagogo-Jack and Shaw (2018) [52]
	2.2. Different types of molecular changes in coding genes	JamalHanjani et al. (2017) [7] Sharma and Debinski (2018) [41] Karachaliou et al. (2015) [40]
	2.2.1 SNV	
	2.2.2 Insertions and Deletions	
	2.2.3 Copy number variation	
	2.2.4 Rearrangements (i.e translocations)	Skoulidis and Heymach (2019) [42]
	2.3. Genomic Instability (CIN and MSI)	Dagogo-Jack and Shaw (2018) [52] Andor et al. (2016) [53]
	2.4. Molecular and biochemical redundancy in the several pathways altered in malignant cells	Logue and Morrison (2012) [54]
	2.5. Heterogeneity at genomic level is not always related with heterogeneity at proteomic level	Ramon Y Cajal S et al. (2017) [55] Ramon Y Cajal S et al. (2018) [56]
3. Epigenetic heterogeneity	3.1 Different changes (histone modifications, DNA methylation) on the genome associated with gene silencing / gene activation	Kumar et al. (2018) [57] Dong et al. (2017) [58] Bhawal et al. (2007) [59]
	3.2. Deregulation of gene expression (overexpression or inhibition)	Agarwal R et al. (2017) [60]
	3.2.1 conding genes: mRNAs	
	3.2.2 non-coding RNAs: miRNAs, lncRNAs	Raychaudhuri et al. (2012) [33] Eriksen et al. (2016) [31] Ramon y Cajal et al.(2019) [34]
	3.3. Associated and associated with the microenvironment	Assenov et al. (2018) [61] Yuan Y (2016) [62]

### Proteomic heterogeneity: going beyond the genome

If the genetic diversity of constitutive alterations in DNA is enormous, then at the level of the proteome, this diversity increases exponentially. Given that proteins are the final effectors of all cellular pathways, along with small metabolites, it seems reasonable to think that the “ideal” targets for therapy are those protein factors that have the most stable expression and activation in tumor cells. Therefore, it is essential to consider the proteomic heterogeneity of tumors.

Even in tumors with constitutive genetic activation of EGFR and HER2, the underlying pathways are not permanently and homogeneously active in all cells [79–83]. At present, approaches such as multispectral imaging of multiple proteins from a common signaling pathway allow the accessible, multiplexed elucidation of proteomic heterogeneity at the level of signal transduction [84]. Moreover, proteomic heterogeneity is not always a simple consequence of the heterogeneity found at the genetic level. In fact, it may be affected by the microenvironment and stress situations such as starving or hypoxia [62, 85].

Much attention has been paid to the role of the molecular pathways controlling RNA splicing [86, 87], the impact of the expression of various protein isoforms, and how the local environmental factors determine their levels [88]. However, protein synthesis machinery and other translation regulators are also significantly modulated by local environmental conditions. Control of protein synthesis (and preceded immediately by regulation of transcription) is considered one of the leading post-transcriptional mechanisms for control of gene expression. This control is profoundly altered in cancer [56].

Alterations in the expression and activity of specific translation factors and their inhibition by cellular stress conditions (e.g. hypoxia or lack of nutrients through various pathways) are common to most human tumors (especially in advanced stages) [56]. The tumor takes control of translation by various mechanisms to cover the demands associated with high proliferation rates or to promote translation of specific messengers that are favorable to tumor progression (survival, pro-angiogenic, invasion, and metastasis) (reviewed in [89]).

## Biological interactions among distinct tumor clones and the microenvironment: the stroma may have a significant impact on phenotypic heterogeneity

Twenty years ago, we published the first histopathologic evaluation of whether differentiation in squamous cell carcinoma could be related to the components of the stroma [90]. After previous studies by Dotto and Weinberg [91], who observed that normal fibroblasts could inhibit the growth of RAS-transformed keratinocytes in athymic mice, we observed that coinjection of normal fibroblasts together with RAS-transformed keratinocytes induced benign or low-grade malignant squamous lesions with extensive areas of keratinization that were observable by morphology, by immunohistochemistry, and by electron microscopy [90]. Subsequently, it was concluded that this fibroblast-mediated differentiation was secondary to factors such as signaling by transforming growth factor beta (TGF-β).

Years of research have shown that the peritumoral stroma in many malignant tumors play an important role and can secrete factors associated with poor prognosis (e.g., chemokines secreted by tumor-associated histiocytes, macrophages or fibroblasts [the so-called cancer-associated fibroblasts]). Signatures of released stromal factors have been thought to affect progression and tumor differentiation, as well as invasiveness in adenocarcinomas [92–95]. Therefore, the importance of the surrounding stroma in intratumoral morphologic heterogeneity seems evident, both regarding factors released by fibroblasts and factors released by inflammatory cells such as histiocytes and lymphocytes. Accordingly, tumor multifocality has been postulated as being associated with the underlying stroma [96]. Morphological and genetic heterogeneity is the result of a multistep process of tumorigenesis that leads to subclonal tumor cell populations with distinct traits, according to current paradigms [2, 5, 8, 21]. The model we present herein incorporates complementary theories of tumor evolution such as the big bang model or the cancer stem cell hypothesis [97] (Fig. 2). It is increasingly clear that understanding alterations within tumor cells is only part of the picture, and we need to understand interactions between tumors and their microenvironment to account for multiple aspects of tumor progression and therapeutic resilience [55, 98–100].

In this regard, the concept of cancer as a consortium of clones and local factors has been proposed [101]. The concept of clonal cooperation is based on a single clone being unable to acquire all the necessary properties to be an invasive tumor, such that various clones must act synergistically and complementarily to acquire the characteristics described by Hanahan and Weinberg [49] and the proposed biochemical changes in 10 or more cellular biochemical pathways. This cell cooperation can be observed in cell clusters in metastatic development: clusters of circulating tumor cells (CTCs) are associated with higher number of metastases than single circulating cells in models of breast cancer, pancreatic cancer, and melanoma [50, 51, 102–106]. Moreover, our group made an effort to generate MDA-MB-231 breast cancer cell lines single clones and demonstrated that the clonal cooperation confers aggressiveness and tumor progression [106].

The microenvironment plays a role in the adoption of phenotypes that may be clinically relevant and are contingent upon the implementation of metabolic gene expression programs and, as such, can be completely independent of the acquisition of new drivers. One of the most explicit examples is the well-known role of the HIF family of transcription factors, which, under hypoxic conditions, trigger a set of adaptive transcriptional responses (tumor angiogenesis, cell metabolism, invasion, survival, therapeutic resistance, and even differentiation and self-renewal) and seem to play a critical role in tumor progression [107].

## How to address tumor heterogeneity in a clinical setting

**Fig. 3:**
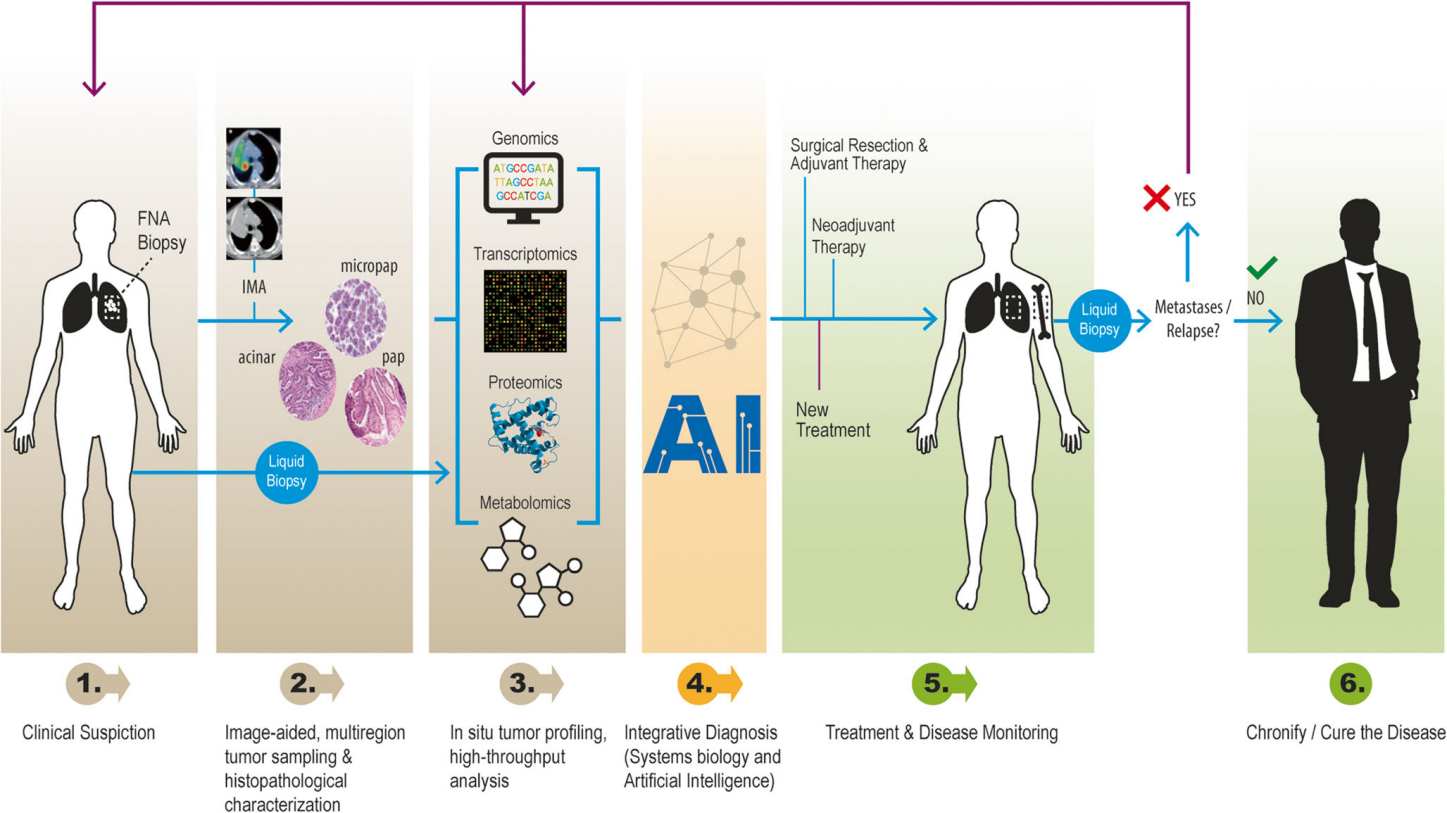
Cancer biology-driven personalized medicine. Schematic representation of the clinical workflow for lung cancer diagnosis, treatment, and follow-up

Intratumor heterogeneity (and its genetic and non-genetic determinants) is a dynamic phenomenon that is observed at multiple levels, and that follows a mainly Darwinian-type progression, although it is far more complicated than previously thought. Unpredictable and often chaotic cellular reactions depending on oncogenic alterations and environmental factors drive tumor progression and hold the key to interpreting tumor development. This concept is essential because the decisions made during a patient’s treatment are based on the study of biopsy specimens of the primary tumor by pathologists and usually revolve around the oncogenic drivers known at the time of diagnosis [2] (Fig. 3). Given the complex and constant development of tumor architecture, it is essential to understand that molecular changes (both genetic and epigenetic) within the tumor itself evolve during disease progression and metastasis [108]. Therefore, the biopsy of a primary tumor is not necessarily predictive of what happens in secondary deposits [2]. In addition, chemotherapy and radiotherapy can trigger selection of resistant clones [2, 109, 110], induce new mutations and other genetic and chromosomal rearrangements [21, 111], recover functionality of previously inactivated genes whose potential had been exploited in synthetic lethal interactions [112], activate cellular dedifferentiation and transdifferentiation programs [97], and even potentiate the development of specific populations by non–cell-autonomous mechanisms [113]. Thus, it is the adoption of both genetic and non-genetic subclonal changes that endows cancer with enough phenotypical plasticity to adapt to microenvironmental pressures and successfully overcome the barriers posed by antitumoral therapy. Otherwise, dissecting tumor heterogeneity involves emerging strategies such as multiregional sequencing, analysis of autopsy samples, single-cell sequencing, and longitudinal analysis of liquid biopsy samples [52]. Rapid research autopsy of cancer patients can explain heterogeneity processes including cancer evolution and acquired therapeutic resistance [114–119].

Numerous studies have shown how genetic variants emerge after therapy and suggest that resistance and response to therapy from that moment onwards are commonly determined by genetic variants (see [8] and references therein). For example, in colon adenocarcinoma, highly sensitive techniques and application of anti-EGFR therapy have made it possible to detect up to 70% of Ras mutations in blood in series where the percentage diagnosed in the primary tumor was approximately 40–45% based on standard molecular techniques [120]. This is also true for non-small cell lung cancer and EGFR mutations [40]. In this sense, therapy has been considered both a source of variability and a selective filter, promoting the acquisition of new mutations and the selective proliferation of previously dormant, minority clones [21, 121].

Given that the strategy of targeting cancer-initiating mutations has been applied with limited success [122], we believe that better comprehension of the determinants of tumor heterogeneity is needed (especially in intratumor terms). Pathologists have the responsibility to make a correct and verifiable diagnosis, from their examination of tissue samples, taking into consideration all the variables that underlie intratumoral heterogeneity. Tumor progression assessment would ideally analyze at least two samples to compare the biologic markers relevant for progression in both tumor cell clones and the microenvironment. The tumor clone markers include those involved in the tumorigenic expansion (proliferation) and invasion, the two leading forces driving progression. The tumor microenvironment analysis focuses the attention on the qualities that potentiate clonal expansion and invasion of tumor cells. In essence, tumor progression analysis must concentrate on clonal heterogeneity and overcome the problems it presents.

One important aspect is how representative biopsies reflects the overall tumor histology and biology. Core biopsies often only reflect a spatiotemporal snapshot of the whole tumor and are therefore unlikely to be fully informative about the clonal composition [123–129]. The size of the sample is another critical issue [130–132], and signal-to-noise ratios need to be balanced. One way to achieve this balance is isolate by microdissection multiple relatively small regions of tumors that more likely represent the balance of morphologically distinct units. The importance of such approach is highlighted by the observation of clustered populations within a tumor that differ in gene expression [133], as well as genetic composition [134]. However, unless large numbers of samples are provided for each tumor, this approach can easily fail to identify patches of genetically distinct cells [130–132]. On the other hand, larger samples, or pools of samples, lead to intermixing of small anatomically distinct units, which provides additional challenges in relation to distinguish distinct functional heterogeneity. Multiple solid biopsy samples should be taken based on data obtained through imaging and nuclear medicine, with the selection of the biopsied area relying increasingly on criteria such as particular metabolic activity. It is also essential to bear in mind that microenvironmental factors such as hypoxia and inflammatory infiltrate can induce changes in the protein expression of therapeutic targets and condition the response to antitumor agents. Therefore, we must select the most representative areas for massive parallel sequencing and genomic and proteomic studies and report on their limitations.

The histopathological diagnosis should integrate molecular analysis (genome sequencing, transcriptome profiling) and protein expression profiling (especially analyses including next-generation sequencing (NGS) techniques) and be able to include gene signatures that are characteristic of a different prognosis or clinical treatment [55, 70, 92, 94, 130, 135–137] (Fig. 3). The incorporation of NGS and the development of new resources for the analysis of these big data, combining molecular and expression signatures, are becoming crucial for diagnosis [13, 138]. The field of radiogenomics, which correlates genomic data with the radiological features of the tumors, must also be taken into account [139, 140]. While this approach based on artificial intelligence may be interesting for the differential diagnosis of radiological features, we understand that genomic information from a single sample is not necessarily representative of the whole tumor and its heterogeneity. While procuring multiple metastatic tumor samples for genomic studies through NGS and development of patient-derived xenografts or organoids, mechanistic insights gained from research autopsy studies of cancer patients can help identify new targets for therapeutic intervention [114]. In collaboration with Cambridge CRUK, our group has performed extensive multi-platform profiling of metastases in 10 warm autopsies of patients with lethal multi-therapy-resistant breast cancers (DNA sequencing, RNA sequencing, the T cell receptor (TCR) sequencing, and immunohistochemistry (IHC)) of multiple individual metastases (range 5–36 metastases per case, 182 individual metastases to 22 organ sites). This collection allowed us to characterize the mutational and copy number aberration (CNA) landscapes across the individual metastasis, to infer the clonal ancestries of metastases, to assess the TME in each individual metastasis, to characterize the predicted neo-antigens, and to assess the TCR repertoires across metastases, providing an unprecedented molecular characterization of lethal breast cancers that had been subjected to multiple lines of systemic therapies [119].

Finally, one of the most powerful techniques is the study of tumor heterogeneity at the cellular level. This approach, called single-cell sequencing, is based on the isolation of dozens of cells in different areas of the tumor, and the study of various Multi(omics) over them [141, 142]. For example, DNA sequencing after gene amplification can allow the study of mutations, amplifications, deletions, and translocations in various areas of the tumor, thus characterizing the homogeneity of these genetic alterations [141, 143, 144]. Expression studies are also done, both at the RNA level (RNA-Seq) and epigenetics with methyloma sequencing [143, 145, 146]. These studies, nowadays, can be expensive and tedious in time as well as in their interpretation, but are already showing results of high clinical interest; for example, the identification of heterogeneity of mutations of the PIK3CA gene in breast cancer with HER2 amplification where the authors describe that PIK13A and HER2 are not always present in the same cells and that chemotherapy selected the cells with mutant PIK3CA [111]. It is an example of the importance of studying intratumoral molecular heterogeneity and where single-cell sequencing technology can be decisive.

## How do we envision cancer research and treatment in the coming years?

1. To assess intratumoral heterogeneity of tumors efficiently, it is essential to systematically integrate molecular patterns, protein expression, and morphology into the fuller context of all clinical and pathological information available (Fig. 3). We have proposed the term *tissunomics*, whereby a diagnosis is individually assessed based upon a combined picture derived from the clinical, pathological, molecular, and protein expression data of the tumor and its surrounding microenvironment [55]. Importantly, molecular diagnosis based on small samples and genetic alterations can lead to a false negative diagnosis or treatment due to genetic and epigenetic changes present in a small subset of tumor cells. In addition, tumor type and location has been shown to underlie unpredictable treatment responses targeting the same molecular pathway, such as the tumor response in melanomas vs. colon carcinomas with BRAF mutations. More conclusive data from basket trials and umbrella trials are needed [55].

Every effort should be made to form multidisciplinary teams involving radiologists, nuclear medicine specialists, pathologists, oncologists, systems biologists, molecular biologists, and data scientists. Tumors must be analyzed at the genetic, molecular, and clinical-radiological level, with integration and correlation of findings to ensure a holistic approach.

(2) To overcome tumor heterogeneity, research should be directed towards the search for central nodes, funnel factors, master regulator genes, and non-oncogene addictions [122, 147–149], in an attempt to confer therapeutic sensitivity. Regarding drug development in malignant tumors and current paradigms in cancer research, new agents include those that target cancer-related vulnerabilities in receptor tyrosine kinases and intracellular signaling pathways, epigenetics, metabolism, and nuclear-cytoplasmic transport, among others. The study of the tumor immune microenvironment appears quite promising and includes treatment with immune checkpoint antibodies, with programmed death 1 (PD-1 and PDL-1) targeted agents, and novel immunotherapies. It is likely that combinations will be needed for most subtypes. Recent studies in solid cancers have highlighted the presence and relevance of immune heterogeneity and that intratumor heterogeneity may also influence the anti-tumor immune responses [150–152].

Several studies [153–160] have shown that the expression of factors such as 4EBP1 and EIF4E is diffuse in most solid tumors and glioblastomas and is associated with lower survival and poorer prognosis. We proposed the concept of *funnel factors* [80], that is, factors that channel crucial information on tumor progression independently of the level at which a specific oncogenic alteration occurs. These factors, which play a significant role in the control of protein synthesis, could be sensitive tumor targets in a large number of malignant tumors [79, 83, 161, 162].

Complex models that implement combinatorial therapy are likely to be particularly beneficial in tumors with a high degree of tumor heterogeneity. In this broad context, evolutionary clues and new findings on interclonal relationships should also be taken into account [81, 101, 113, 163]. The identification of factors involved in this interplay between malignant clones, which mediate tumor growth and metastasis, may be one promising approach in the understanding of cancer [101]. Therefore, studies carried out from the perspective of systems biology [149], tailored towards the identification of hubs or other central factors in this complicated tangle of biochemical networks responsible for maintaining the tumorigenic state, will be fundamental in the identification of addictions and vulnerabilities in cancer that would otherwise be difficult to imagine [147, 164].

(3) Liquid biopsies. Difficulties in obtaining tumor tissue using invasive surgical procedures have led to the development of liquid biopsies for several cancer types [165–184]. They comprise tumor-derived nucleic acids (e.g., circulating cell-free tumor DNA [ctDNA], microRNA), circulating tumor cells (CTCs), and tumor-derived extracellular vesicles that accumulate in the blood, cerebrospinal fluid (CSF), urine, saliva, and other fluids [165, 178, 185–191]. One advantage of liquid biopsies is that it significantly reduces the problem of spatial heterogeneity. Several studies, comparing blood and tissue biopsies, have confirmed that this approach has high specificity, although variable sensitivity is reported. Another important advantage (although under certain situations it may be a disadvantage) is that it tends to reflect an aggregate of the output (ctDNA/CTC etc.) potentially from both primary and various metastatic sites. Such complex tumor heterogeneity cannot be evaluated by a single core tumor needle biopsy [192].

However, the most clinically advanced approach is ctDNA from plasma which closely matches the gene profile of tumor tissue biopsies. Plasma ctDNA provides tumor-derived material to identify actionable genomic alterations, monitor treatment responses, predict progression of the tumor before clinical or radiological confirmation, and can identify mechanisms of resistance also during therapy [173, 174, 176, 193, 194]. For a comprehensive review, see [195].

Prospective clinical studies using liquid biopsies have characterized and monitored over time the genomic alterations of patients [40, 174]. Recently, the TRACERx consortium [7, 196] investigated tumor heterogeneity and evolution in early-stage NSCLC and showed the prognostic value of copy-number heterogeneity assessment in tumor biopsies and circulating tumor DNA detection in plasma. However, these liquid biopsy results reflect a kind of summary of tumor burden, regardless of the origin of the tumor cells (from primary or metastatic deposits), and require some degree of by-pass of microanatomical boundaries (vascular basement membrane and stromal invasion) by either active tumor invasion or passive external damage (e.g., ischemic or inflammatory). In this context, some caution should be taken for the evaluation of early epithelial neoplasms.

The role of subclonal driver events in response to therapy and disease recurrence and progression remains to be determined. The use of liquid biopsies may pave the way for a more detailed, real-time patient-tracking approach allowing the modification of therapeutic strategies throughout the disease.

(4) Artificial intelligence. Intratumor heterogeneity is one of the main reasons for the lack of diagnostic reproducibility between pathologists given the complexity of the microscopic interpretation of certain tumors. Furthermore, many biomarkers do not have an established interpretation algorithm. It is critical to improve existing algorithms for the quantification of immunohistochemical and other in situ biomarkers. The development of artificial intelligence algorithms with automatic learning (“deep learning”) is already shaping the field. Deep learning methodology, with the generation of thousands of clinical-pathological diagnostic cases, can promote the development of algorithms based on this methodology that could represent a breakthrough in the pathological diagnosis As an example, Google released TensorFlow, an algorithmic development framework for distributed computing, to the general scientific and technical community. This open-source machine learning tool is free for any qualified scientist and is specialized in cognitive computing.

With this approach, software is being developed by many startups and educational institutions as well as big companies such as Google, Phillips and Leica Microsoft. Algorithm-related applications for primary diagnosis, intraoperative diagnosis, training, quantification of immunohistochemistry, or diagnostic consultation are likely to progress significantly over the next few years. Notably, there have been several claims that the accuracy and reliability of diagnoses based on neural network systems is very high [197, 198]. Examples have been published for skin cancer (both melanoma and squamous cell carcinoma), lung adenocarcinoma, glioma, gastric carcinoma, and others [135, 199–202].

Moreover, the deep learning tumor prediction heat map can be quite complementary to pathologists’ “workflow.” An algorithm can detect, for example, metastatic carcinoma in lymph nodes, or tumor budding in the colon or cervix, and help to recognize histologic patterns associated with higher malignant grades in gliomas [203], and moreover, can score the degree of malignancy in tumors such a prostate adenocarcinomas where quantification of the histological patterns are underway [197, 198].

These algorithms are likely to help pathologists in reaching a faster, more accurate diagnosis and significantly reduce the pathologist-dependent discordance in histopathological diagnosis.

(5) As a final reflection, we firmly believe that research strategies should be optimized. At present, most research teams are small, self-managed groups. Consequently, research is slow, and financial and human resources are not optimized. We must establish more rational and ambitious organizational models and strategies, with real networks and professional, well-trained teams. As Horning recently said [204], “science and technology are at an inflection point with convergence—the integration of life sciences, physical sciences, mathematics, engineering, and information technology—poised to make significant progress”. We must look forward and not forget that our primary objective is to cure cancer or at least make it a chronic disease. Such a social commitment requires us to search for all possible methods of cooperation among those involved in the diagnosis and treatment of cancer.
